# Xylem sap collection and extraction methodologies to determine *in vivo *concentrations of ABA and its bound forms by gas chromatography-mass spectrometry (GC-MS)

**DOI:** 10.1186/1746-4811-8-11

**Published:** 2012-03-22

**Authors:** Andrew G Netting, Julian C Theobald, Ian C Dodd

**Affiliations:** 1 School of Biotechnology and Biomolecular Sciences, University of New South Wales, Sydney, NSW 2052, Australia; 2 Brancott Estate Winery, PernodRicard New Zealand, Blenheim, PO Box 331, New Zealand; 3 The Lancaster Environment Centre, Lancaster University, Lancaster LA1 4YQ, UK

**Keywords:** ABA, ABA-GE, *flacca*, GC-MS, *notabilis*, Mutant, *sitiens*, Tomato, Xylem sap

## Abstract

**Background:**

Accurate quantification of xylem sap ABA concentrations is important to underpin models of root-to-shoot ABA signalling to predict the physiological effects of soil drying. Growing tomato plants in a whole plant pressure chamber allowed sequential xylem sap collection from a detached leaf, the petiole stub of an otherwise intact plant and finally the de-topped root system of the same plant, to determine the impact of xylem sap sampling methodology on xylem ABA concentration. Since xylem sap can contain bound forms of ABA, a novel gas chromatography-mass spectrometry (GC-MS) procedure was developed to chemically separate free ABA from two *in planta *bound ABA forms known as Adducts I and II and ABA-glucose-ester (ABA-GE).

**Results:**

Xylem sap ABA concentrations were highly dependent on the sampling methodology used: the highest concentrations were detected in sap collected by applying an overpressure to detached leaves following the measurement of leaf water potential. Irrespective of xylem sap source, the wild-type cultivars Ailsa Craig and Rheinlands Ruhm had higher free ABA concentrations than a range of ABA-deficient mutants (*notabilis, flacca *and *sitiens*). However, in the mutants, concentrations of bound forms of ABA were similar to wild-type plants, and similar to free ABA concentrations.

**Conclusions:**

Although xylem concentrations of these bound ABA forms and ABA-GE suggest they have a limited physiological impact on ABA homeostasis in tomato, the methods developed here will allow a more complete understanding of ABA biochemistry and root-to-shoot signalling in species known to have higher concentrations of these compounds.

## Background

Roots in drying soil produce chemical signals such as abscisic acid (ABA) [1] that can be transported to the shoots to modify their physiology. While this remains an attractive hypothesis, extensive studies have demonstrated that this signal can be modified by synthesis and metabolism of ABA along the transport pathway [2], and the fundamental basis of root-sourced ABA signalling has been challenged. Reciprocal grafting studies of wild-type (WT) plants and ABA-deficient mutants [3-5] have generally concluded that stomatal closure is independent of root-synthesised ABA since stomatal conductance of WT scions decreased as the soil dried, independently of whether they were grafted on a WT or ABA-deficient rootstock [3]. This closure occurred in plants where shoot turgor was maintained (by root pressurisation) as the soil dried, seemingly excluding a role for local induction of ABA synthesis in the leaves due to decreased turgor [3]. Instead, this suggests that a chemical signal from the roots may increase the apoplastic ABA concentration in the leaves to cause stomatal closure. In WT scions grown on an ABA-deficient rootstock, it is possible that water stress could induce synthesis of bound forms of ABA known as Adducts I and II [6,7] in the roots. These compounds could be transported in the xylem to the shoots where they would release ABA in the xylem sap of the leaves, particularly as the soil dries.

The existence of three ABA-deficient mutants of increasing severity (*notabilis, flacca *and *sitien*s) in tomato provided an opportunity to determine whether these mutants differed in biosynthesis of these adducts. The widely accepted pathway for ABA biosynthesis [8] commences with the oxygenation of 9'*cis*-neoxanthin or 9-*cis*-violaxanthin to give the C-15 aldehyde, xanthoxin. This reaction is under the control of the rate-limiting enzyme 9-*cis*-epoxycarotenoid dioxygenase (NCED). The *notabilis *mutation was identified as a null allele of the LeNCED1 gene containing a single A/T base pair deletion [9]. Next, xanthoxin is reduced to ABA-aldehyde, and then oxidised to ABA [8]. As the *flacca *mutation in tomatoes has an out-of-frame deletion in a conserved C-terminal region of a molybdenum cofactor (MoCo) sulfurase [8], it is likely that all of the constituent aldehyde oxidases are affected. Recently, the *sitiens *gene was identified as an aldehyde oxidase apoenzyme [10], completing the genetic identification of all three tomato ABA-deficient mutants. Since the role of adduct biochemistry in modulating ABA concentrations in these mutants remains obscure, adduct concentrations in xylem sap collected from these mutants was determined.

ABA conjugates, predominately ABA glucose ester (ABA-GE), occur in the xylem sap of several plants [11] and the intercellular washing fluid of barley primary leaves contains a β-glucosidase activity [12] that releases ABA from ABA-GE in the leaf apoplast. The activity of these β-glucosidases increased with salt stress [12] so that ABA-GE is probably a source of stress-induced apoplastic ABA. Consequently, it is important to distinguish ABA-GE and the adducts as sources of free ABA.

Two bound forms of ABA (termed Adducts I and II) were previously characterised in a series of experiments [6,7]. Adduct I contains a xanthoxal-like moiety with substituent peptides (probably tetrapeptides) at C-4' and C-1 positions of the ABA molecule. The peptide at C-4' is attached as an *enol-*ester and contains a histidyl residue and possibly a positively charged amino acid while the peptide at C-1 is attached via the aldehyde group to give a hemiaminal with the N-terminal amino group and contains two histidyl residues. In addition, C-1 is substituted with a methoxy group as a hemiacetal. Adduct II contains an ABA-like moiety with the same substituent peptide as Adduct I at C-1 and no peptide at C-4'. The substituent peptide and methoxy group are so attached that an imine ester results. Experiments that exposed detached tomato shoots to ^2^H_2_O and ^18^O_2 _indicated that these compounds can be synthesised in the shoot [7], but their occurrence in xylem sap seems restricted to a solitary (unpublished) report (AG Netting and RE Munns, unpublished results). Further work is required to definitively establish the pathways of adduct biosynthesis and subsequent catabolism *in planta *to liberate free ABA.

**Figure 1:**
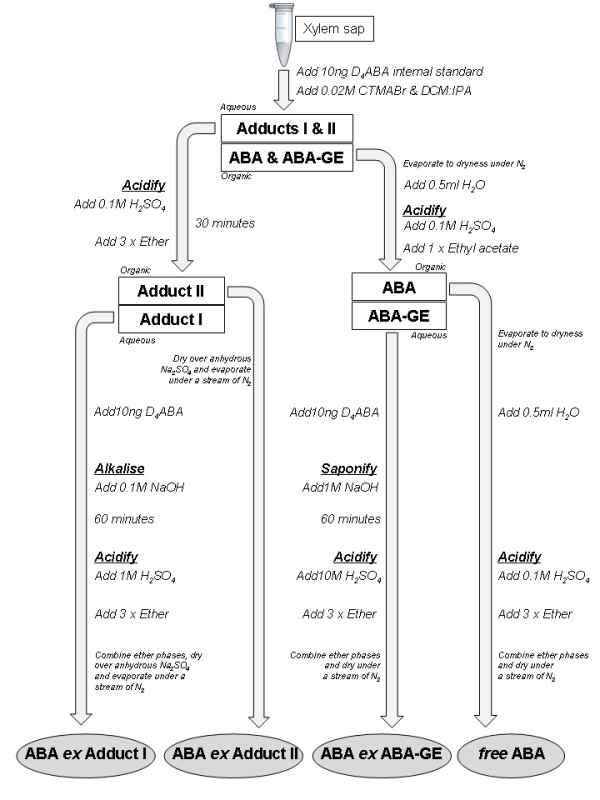
**Schematic outlining the separation of ABA, ABA-GE and Adducts I & II from xylem sap, and their release as ABA *ex *Adduct I, ABA *ex *Adduct II, ABA *ex *ABA-GE and *free *ABA, prior to subsequent derivatisation to pentafluoro-benzyl esters (PFB-ABA) (not shown but see text) for quantification by GC-MS**. Horizontally paired rectangles represent key phase separations and are denoted as aqueous or organic accordingly. Abbreviations used: ABA, abscisic acid; ABA-GE, abscisic acid-glucose ester; D_4_ABA, tetra-deuteratedabscisic acid; CTMABr, cetyltrimethylammonium bromide; DCM, dichloromethane; IPA, *iso*-propanol.

**Figure 2:**
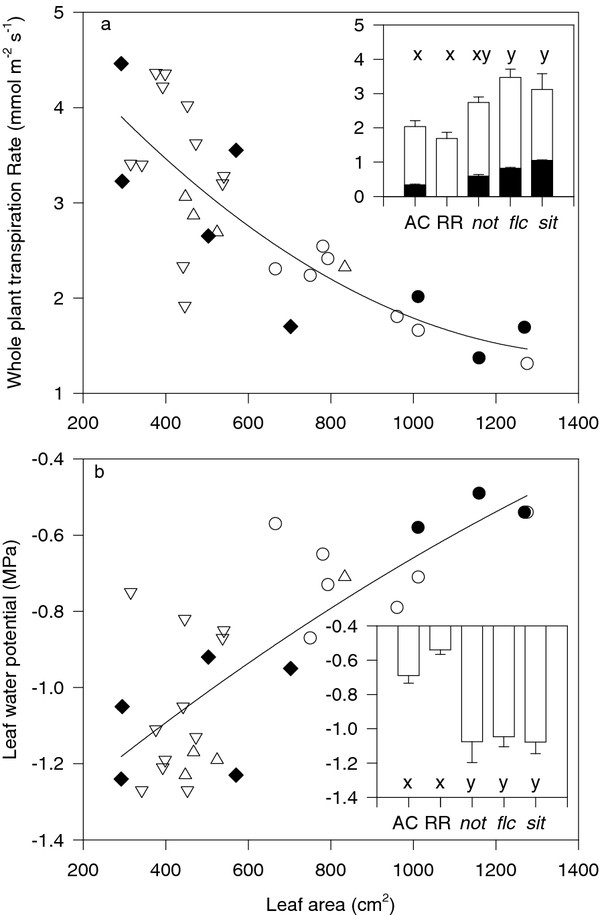
**Sap collection methodology**. Diagrams at top indicate the experimental sequence of (1) leaf removal to allow measurement of Ψ_leaf _in a Scholander pressure chamber (applied pressure is equal, but opposite in sign, to Ψ_leaf_), followed by (2) application of an overpressure to collect xylem sap from the detached leaf, (3) sealing the plant into the whole plant pressure chamber to collect xylem sap from the petiole stub by pressurising the root system, (4) removal of the whole shoot while the root system is under pressure to allow (5) xylem sap collection from the root system. Red bars in 1 and 4 indicate sites of organ excision. Applied pressure (**a**), sap flow rate (**b**) and xylem ABA concentration (**c**) of sequential samples collected from a typical AC plant. Each point is a separate measurement. Vertical dotted lines discriminate the three sources of xylem sap collected. Horizontal arrows in (**b**) indicate average whole plant transpiration rate over the 3 hours prior to leaf excision, and estimated leaf transpiration rate (assuming proportionality between transpiration rate and leaf area). Vertical arrows in (**b**) and (**c**) indicate mean (± SE) values of sap flow rate and xylem ABA concentration for the samples enclosed by a box, where SE is smaller than the symbol.

To determine the relative proportions of ABA-GE, adducts and free ABA in tomato xylem sap, a novel GC-MS procedure was developed to allow chemical separation of these components (Figure 1). Tomato was chosen for these experiments due to the availability of several ABA-deficient mutants, and since it contains appreciable quantities of conjugated ABA in the xylem sap [13]. Growing plants in specialised pressure pots permitted sequential xylem sap collection from detached leaves (after leaf water potential was measured in a Scholander-type pressure chamber, an overpressure was applied to collect sap), the remaining petiole stub and finally the de-topped root system by enclosing and pressurising the entire root system (Figure 2). Accurate quantification of xylem ABA concentrations from both leaves and roots is important to verify the accuracy of models of ABA root-to-shoot signalling in order to understand physiological impacts of different irrigation techniques [14-16] that aim to increase shoot ABA status to increase crop water use efficiency. Thus this work aimed to extract, purify and quantify ABA ex Adduct I and II, determine their xylem sap concentrations in mutants differing in endogenous ABA titres, and to determine the most suitable method to collect xylem sap to study root-to-shoot signalling.

## Results

**Figure 3:**
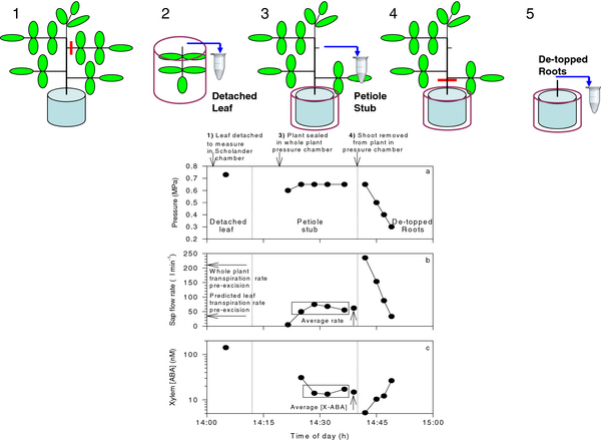
**Whole plant transpiration rate (a) and leaf water potential (b) of all tomato plants harvested in this study plotted against whole plant leaf area, where each point is a separate AC (hollow circles), RR (filled circles), *not *(triangles), *flc *(inverted triangles) and *sit *(filled diamonds) tomato plant**. Lines are second order regressions. Insets indicate means ± SE of 7 (AC), 3 (RR), 4 (*not*), 11 (*flc*) and 5 (*sit*) replicates, with different letters above the bars indicating significant (P < 0.05) differences according to Tukey's HSD test. Filled bars in inset (**a**) are re-plotted from [17], where all the ABA-deficient mutants were available in the AC background.

Since wild-type plants grew faster than the ABA-deficient mutants, the latter had a smaller leaf area when harvested. Consequently whole plant transpiration rate (E) apparently decreased, and leaf water potential (Ψ_leaf_) increased as whole plant leaf area increased (Figure 3). Despite this developmental confounding and considerable inter-plant variation, it was apparent that E was lower and Ψ_leaf _higher in the wild-type Ailsa Craig (AC) and Rheinlands Ruhm (RR) plants (Figure 3 insets). In contrast to previous work where whole plant transpiration rate generally increased with increasing severity of ABA deficiency [17], there were no significant differences between the ABA deficient mutants (Figure 3a inset). Likewise, Ψ_leaf _was similar between the ABA-deficient mutants (Figure 3b inset).

The pneumatic pressure applied to both Scholander (to measure Ψ_leaf_) and whole plant (to collect sap from the petiole stub, and then the de-topped root system) pressure chambers are plotted (Figure 2a), along with the sap flow rates and xylem ABA concentrations (measured by radioimmunoassay - RIA) in a typical AC plant. Initially, a leaf of 131 cm^2 ^(16.5% of total plant leaf area) was detached to measure leaf water potential (-0.73 MPa) and xylem ABA concentration (142 nM) (Figure 2a, c). Applying 0.6 MPa to the roots resulted in negligible sap flow (5 μL min^-1^) from the remaining petiole stub (Figure 2b), much less than that calculated to occur through that petiole prior to leaf excision (35 μL min^-1^), assuming that whole plant transpiration (207 μL min^-1^) was evenly distributed across total plant leaf area. Accordingly, the pressure applied to the root system was increased to 0.65 MPa, which increased sap flow by an average of 12.5-fold (± 20%) during the rest of the experiment (Figure 2b). Xylem ABA concentration of the initial sap sample collected from the petiole stub was 31 nM, but this declined and remained relatively stable in sequential samples (14.8 ± 1.1 nM, n = 3). Excising the entire shoot while the plant was under pressure (0.65 MPa) resulted in a high sap flow rate (235 μL min^-1^), which decreased as the pressure was decreased in 0.1-0.15 MPa decrements. Xylem ABA concentration in samples collected from the de-topped root system was inversely related to sap flow rate (cf. Figure 2b, c), with a maximum of 26 nM in the slowest flowing sap and a minimum 5 nM in the fastest flowing sap.

**Figure 4:**
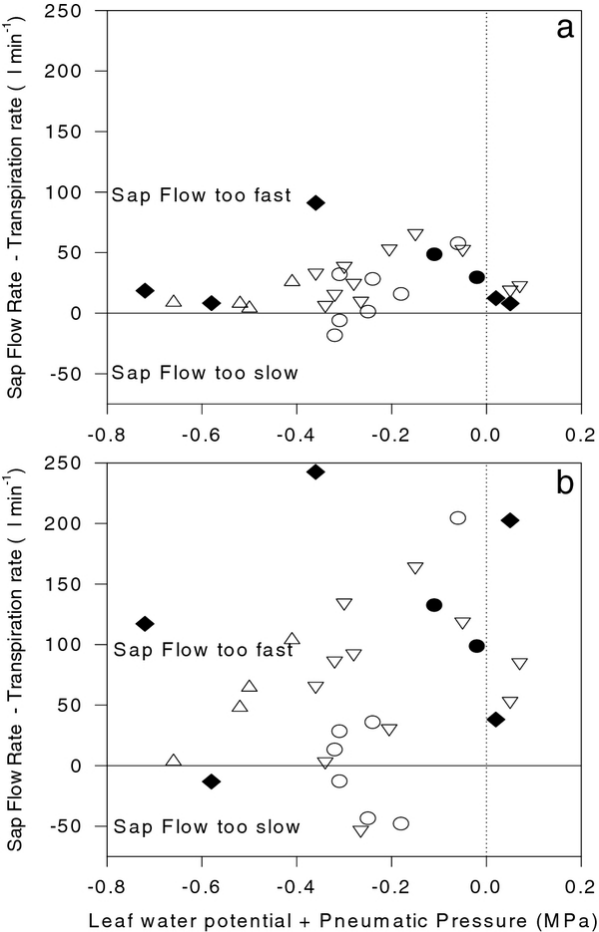
**Difference between pressure-induced sap flow rate from the petiole stub and estimated leaf transpiration rate (a) and pressure-induced sap flow rate from the de-topped root system and whole plant transpiration rate (b) plotted against the difference between leaf water potential and plant balancing pressure**. Each point is a separate AC (hollow circles), RR (filled circles), *not *(triangles), *flc *(inverted triangles) and *sit *(filled diamonds) tomato plant.

For each plant, pressure-induced sap flow rates from the petiole stub and de-topped root system were compared with estimated *in vivo *leaf or whole plant transpiration rates respectively by calculating the difference. This was plotted against the difference between leaf water potential and the applied pneumatic pressure (Figure 4). Generally, the pneumatic pressure applied to the root system that enabled sap flow from the petiole stub was much less than that applied to the detached leaf to measure water potential. Despite this, sap flow rates from the petiole stub were 2.1-fold higher than transpirational flow rates (assuming that leaf transpiration rate was proportional to leaf area) while root xylem sap flow rates were 1.45-fold higher than whole plant transpirational flow rates, when averaged across all plants measured in this study.

**Figure 5:**
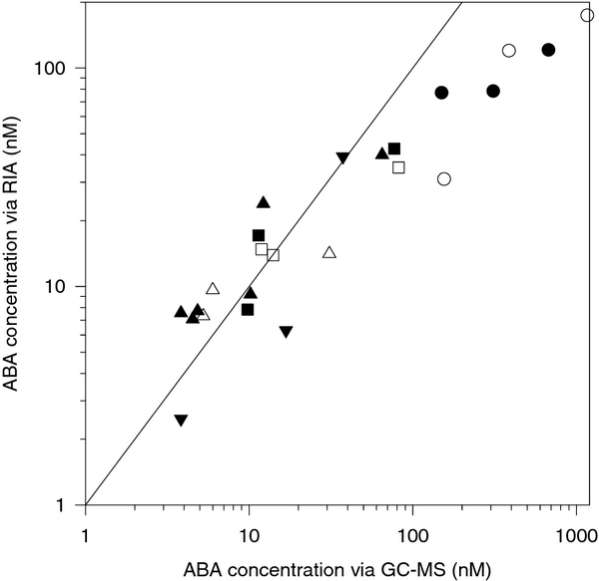
**Comparative quantification of ABA precursors, ABA and ABA-GE using GC-MS and RIA techniques for free ABA (circles) ABA *ex *Adduct I (triangles), ABA *ex *Adduct II (inverted triangles) or ABA *ex *ABA-GE (squares) for xylem sap collected from the roots (filled symbols) or the petiole stub of otherwise intact tomato plants (hollow symbols)**. Each point is a separate xylem sap sample, with the solid line representing the 1:1 relationship.

In a separate group of AC plants grown over a much wider range of soil water contents and Ψ_leaf _(0.18 to 0.33 g g^-1 ^and -0.86 to -1.16 MPa respectively), comparative quantifications of ABA and related compounds using GC-MS and RIA techniques (using the same sample preparation and handling method for both) gave similar results when concentrations were less than about 40 nM (Figure 5). At higher ABA concentrations, the RIA response saturated, possibly due to one or more of the organic chemicals (used in separating the four possible sources of ABA) interfering with ABA:antibody binding. Consequently, all subsequent measurements used GC-MS techniques.

**Figure 6:**
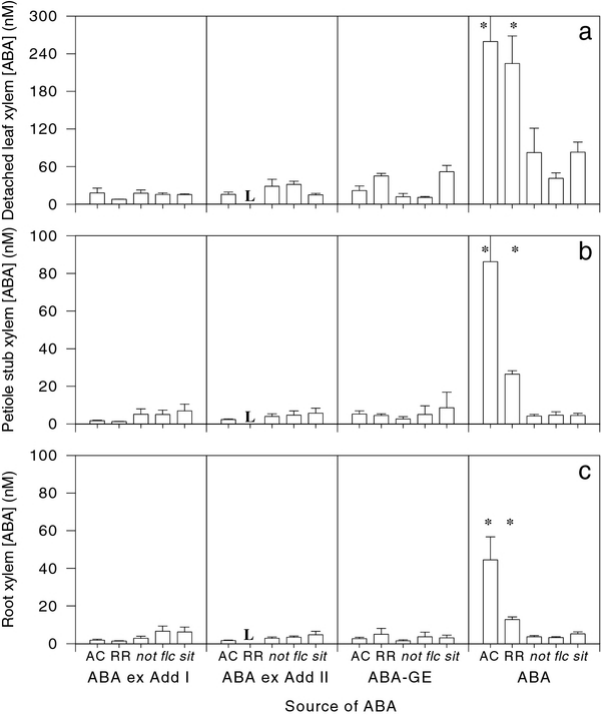
**Concentrations of ABA precursors, ABA and ABA-GE and metabolites in sap collected from detached leaves (a), the petiole stub of otherwise intact tomato plants (b) and the root system (c)**. Note the change of y-axis scale in (a). Data are means ± SE of 2-5 replicates, with * indicating that concentrations were significantly (P < 0.05) different from other values in each panel. L indicates that samples were lost during the extraction process.

Xylem sap collected from detached leaves had the highest concentrations of free ABA and other compounds compared to sap collected from the remaining petiole stub or the de-topped root system (cf. Figure 6a-c). Although AC and RR (wild-type) plants had higher levels of free ABA than the ABA-deficient mutants, irrespective of the source of xylem sap, there were no significant genotypic differences in concentration of the ABA adducts or ABA-GE.

**Figure 7:**
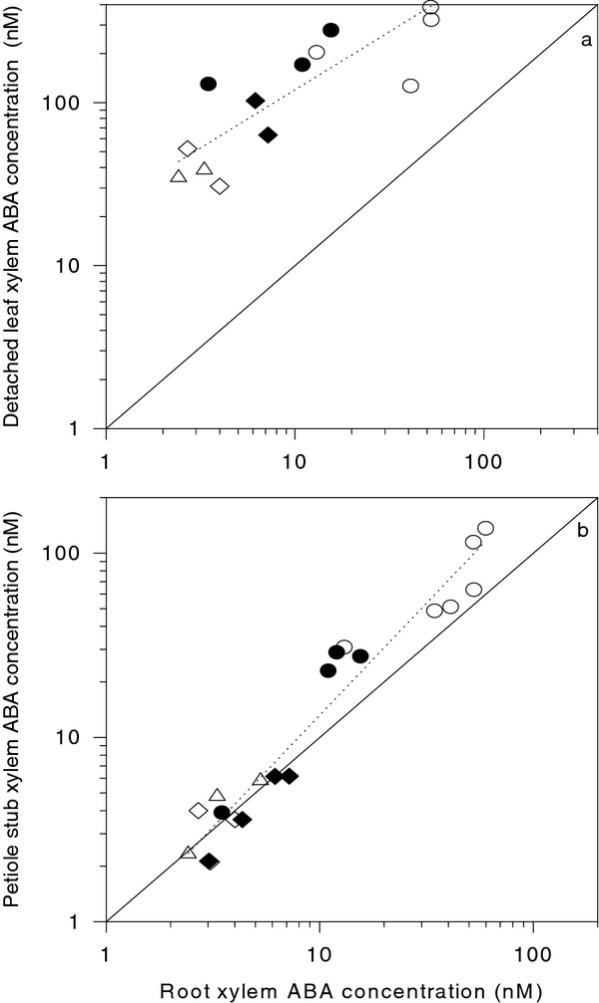
**Xylem ABA concentration of sap collected from detached leaves (a) and the stub of the same petiole via root pressurisation (b) plotted against root xylem ABA concentration for AC (circles), RR (filled circles), *not *(triangles), *flc *(inverted triangles) and *sit *(filled diamonds) tomato plants**. Each point is a separate xylem sap sample, with the solid line representing the 1:1 relationship, and the dotted line indicating the regression between the points.

As reported previously [18], xylem sap collected from detached leaves had appreciably higher ABA concentrations than root xylem sap (Figure 7a). Collection of xylem sap from the petiole stub gave ABA concentrations similar to those determined in root xylem sap (Figure 7b). However in WT (AC and RR) plants, ABA concentrations in sap samples collected from the petiole stub were approximately twice those of samples collected from the roots.

## Discussion

Ongoing interest in irrigation techniques such as partial rootzone drying has stimulated the measurement and modelling of *in planta *xylem ABA concentrations [14-16]. Nevertheless, methodological difficulties associated with xylem sap sampling can potentially devalue such information. In field-grown crops, root xylem sap can only be sampled by de-topping the plant and collecting sap at relatively low flow rates (compared to whole plant transpiration rate), which artificially increases xylem sap ABA concentration (cf. Figure 2b, c), as previously observed [19]. Consequently, several studies have collected xylem sap samples by detaching leaves or stems, measuring their water potential and then applying an overpressure to collect sap (e.g. [18,20]). While varying the overpressure applied to detached tomato leaves seems to have a minimal effect on the ABA concentration of the xylem sap collected [5], actual concentrations can be higher ([18]; Figure 7a here) than root xylem ABA concentration, depending on both the accuracy with which root xylem sap flow rate is matched with transpirational flow rate (Figure 2b;4b) and/or an augmentation of leaf apoplastic sap with symplastic contents during sap collection [20]. For this reason, attempts to model xylem ABA concentrations of plants exposed to heterogeneous soil moisture may be more informative when a single xylem sap sampling methodology and/or site of xylem sap sampling (either root or leaf) is adopted.

Using the whole plant pressure chamber to vary sap flow rate can provide a precise estimate of *in vivo *ABA concentrations (at equivalent flow rates to transpiration) and/or evaluate the sensitivity of xylem ABA concentration to sap flow rate. One of the difficulties of collecting a leaf xylem sap sample that is representative of the transpiration stream is that any removal of tissue decreases hydraulic resistance, thus xylem sap can potentially flow at a higher rate than transpiration [21]. Nevertheless, minimising the leaf area removed (relative to total plant leaf area) may allow xylem sap to flow from petiole stubs at rates approximating *in vivo *transpiration rate [22]. Here, although pneumatic pressures applied to the roots were generally numerically less than leaf water potentials, flow rates from the petiole stub were generally higher than predicted leaf transpiration rates (Figure 4a), which may have underestimated *in vivo *ABA concentrations. Consequently, more advanced xylem sap sampling systems have been developed, that involve lowering the humidity around the leaf prior to sap sampling [23] and/or the use of electro-optical sensors [21] to precisely regulate pneumatic pressures to the root system to match sap flow rates with *in vivo *transpiration rates determined prior to sampling. A further complication is that the first xylem sap sample collected from the petiole stub usually had a significantly higher ABA concentration (Figure 4c), even though the cut surface was washed (with distilled water) and blotted prior to collection. A similar phenomenon was noticed after washing and blotting de-topped tomato root systems, which increased xylem ABA concentration 5-fold [19]. Thus the first sample collected from the petiole stub was excluded when determining ABA concentration from this source of sap.

Although this phenomenon likely also affected determinations of ABA concentration in sap flowing from the root system, removal of the whole shoot while the root system was pressurised resulted in sap flow rates that greatly exceeded whole plant transpiration rate (Figure 4b) as previously reported [24]. This required sequential decreases in pneumatic pressure (Figure 2a) to collect root xylem sap at flow rates approximately equivalent to whole plant transpiration, for further analysis of ABA and related compounds. Nevertheless, in WT plants, ABA concentration in root xylem sap was numerically less than in sap collected from the petiole stub (Figure 7b) due to both the diluting effects of higher flow rates from the de-topped root system (Figure 4b) and/or ABA enrichment of xylem sap as it ascends the plant [13,22]. To gain an authentic sample of the transpiration stream, we advocate xylem sap collection via pressurizing the roots of an "almost intact" plant (removing a minimum amount of leaf tissue is necessary to allow sap flow from the cut surface) grown, or placed in, a whole plant pressure chamber, but note that this technique is not possible in the field !

**Figure 8:**
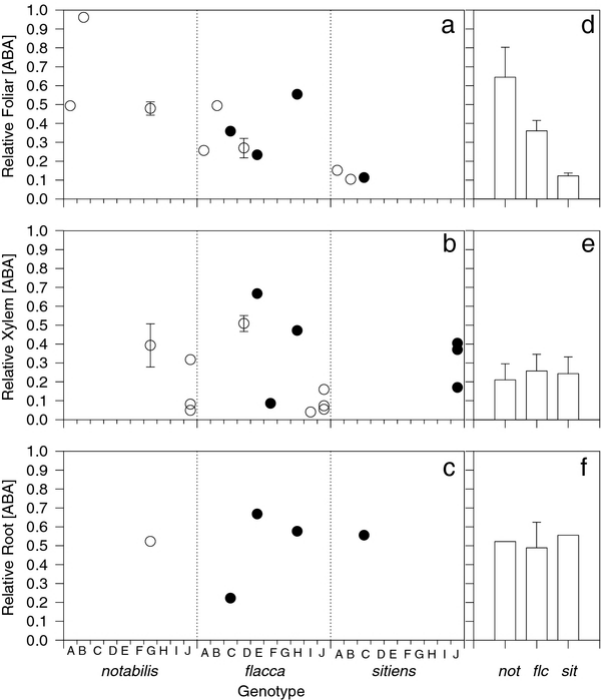
**Relative leaf (a), xylem sap (b) and root (c) ABA concentration of the ABA-deficient tomato mutants used in this study where 1 is the concentration reported for the respective WT plants**. Data re-plotted from [26] (A), [27] (B), [28] (C), [29] (D), [30] (F) [31] (G), [25] (H), [5] (I) and the current study (J) where hollow and filled circles represent mutants in the AC and RR background respectively. Mean data are plotted (without error bars) except where ABA concentrations were measured on more than one occasion or with more than one treatment. Data in a-c are used to calculate the means ± SE shown in d-f.

Irrespective of where or how xylem sap was collected, there were clear genotypic differences in xylem ABA concentration between WT and ABA-deficient mutants (Figure 6), yet all ABA-deficient mutants had similar xylem ABA concentrations. Considerable between-plant variation (Figure 3) and limited replication of some genotypes may have accounted for the similarity of xylem ABA concentration (Figure 6), leaf water potential and whole plant transpiration rate (Figure 3) within the mutants, since a meta-analysis of the literature shows a pronounced gradient in foliar ABA deficiency (Figure 8a, d). Interestingly, genotypic differences in whole plant transpiration rate (Figure 2a - [17]) were seemingly more closely related to leaf ABA concentration (Figure 8a, d) than either xylem (Figure 6; 8e) or root (Figure 8f) ABA concentration, apparently reinforcing conclusions from reciprocal grafting experiments with WT and ABA-deficient tomatoes that the rootstock has little effect on shoot physiology [3,5,25].

Nevertheless, the root system may also supply bound forms of ABA to the shoot, which may be metabolised to release free ABA. The xylem sap of certain species can contain high concentrations of conjugates (in some cases 10-fold higher than free ABA concentrations), especially ABA glucose-ester (ABA-GE), which can be cleaved by apoplastic glucosidases in the leaves to release free ABA [11]. However, irrespective of the source of xylem sap, ABA-GE concentrations were relatively low compared to the concentrations of free ABA in WT plants, but comparable to ABA concentrations in ABA-deficient mutants (Figure 6).

While ABA-deficient mutants may accumulate precursors of ABA such as 2-trans-ABA alcohol [32], there was no evidence from the experiments reported here that the mutants accumulated significant quantities of ABA adducts. Consequently, there is no direct evidence from these experiments that these adducts are, in fact precursors of ABA. However, when these compounds were first discovered [6], it was reported that the adduct components could not be labelled with (±)-[2-^14^C]ABA, (±)-Me[2-^14^C]ABA or with (±)-[^2^H_3_]AB-aldehyde suggesting that the adducts could be precursors of ABA. Furthermore, when tomato seedlings were grown in ^2^H_2_O for ten days with daily wilting [7], the specific activity of deuterium in the adducts was higher than in the ABA isolated from the same plant, suggesting that the adducts are indeed precursors of ABA. Similarly, this result was supported by an experiment in which tomato shoots were incubated in ^18^O_2 _and the adduct components isolated [7]. Since the incorporation of ^18^O into the -COO- group of MeABA derived from Adduct II was some four times the incorporation of ^18^O into the carboxyl of free ABA, this again suggested that Adduct II is a precursor of ABA. Ultimately, precise placement of these compounds within known ABA metabolic pathways will require determination of their complete structures using techniques such as LC-MS (liquid chromatography -mass spectrometry).

Although the ^18^O_2 _labelling experiments with detached tomato shoots described above clearly demonstrated the capacity for tomato shoots to synthesise Adducts I and Adduct II [7], it was unknown whether these compounds could be synthesised in the root system and act as long-distance signals of soil conditions. Previous experiments showed that xylem sap adduct concentrations dramatically increased during the afternoon in mildly water-stressed barley plants (AG Netting and RE Munns, unpublished results) Although Adducts I and II were detected in tomato xylem sap, irrespective of where the sap sample was collected from (Figure 6), their site of synthesis remains unknown since there can be considerable basipetal phloem flows of compounds such as ABA which can be recycled in the root system via the xylem [2]. The relatively low adduct concentrations in tomato xylem sap (Figure 6) suggests that tomato roots are not a significant source of bound forms of ABA to the shoot.

## Conclusions

In species which contain considerable quantities of Adducts I and II [33], the technique described below allows chemical separation of these compounds from both free ABA and ABA glucose-ester to allow their quantification. Although this method was developed using the relatively clean matrix of tomato xylem sap, allowing relatively short preparation and clean-up times, with modification it could be applied to different tissue types and species.

Determining the significance of root-to-shoot transport of these bound forms of ABA to foliar ABA homeostasis requires accurate matching of *in vivo *transpiration rates and pressure-induced sap flow rates, which was conveniently achieved here by growing plants in pots that could be inserted into a whole plant pressure chamber to facilitate sap collection. Significant impacts of xylem sap sampling methodology on actual sap ABA concentrations requires that accurate models of root to shoot ABA signalling use a single sap collection methodology.

## Methods

### Plant culture

Tomato (*Solanum lycopersicum *Mill. cv. Ailsa Craig - AC and cv. Rheinlands Ruhm - RR) and the ABA-deficient mutants *notabilis, flacca *(AC background) and *sitiens *(RR background) were grown in a walk-in controlled environment chamber at the Lancaster Environment Centre. Plants were grown under a 9 h photoperiod, with air temperature ranging from 20 to 26°C, relative humidity from 37 to 61% and a PPFD at plant height of 250 μmol m^-2 ^s^-1^.

Plants were grown in a loam-based substrate (J. Arthur Bower's, UK), in 1.27 L plastic cylindrical pots (200 mm height, 90 mm diameter) capped with an aluminium plate, designed to fit in a pressure chamber. Seeds were initially placed on distilled water-moistened filter paper (Whatman #1) in the dark for 48 hours, prior to transplanting uniform seedlings into substrate-filled funnels that directed root system growth through a hole in the top of the aluminium plate. Ten days after transplanting, soil in the funnels was gently washed away, and plants were allowed to grow for 4 weeks prior to sealing into the top of the pots with silicone rubber (Sylgard 184, Dow Corning, Midland, USA) prior to xylem sap collection. Plants were periodically watered (to replace transpirational losses) throughout development.

Xylem sap was collected between 1 and 6 h after the start of the photoperiod. For several (typically 2-3) hours preceding xylem sap collection, whole plant transpiration rate was determined by weighing the plant hourly. A fully expanded leaf was removed from the shoot to measure leaf water potential using a Scholander-type pressure chamber. An overpressure was applied (for 1-2 minutes) to the leaf to collect xylem sap as previously described [18] then leaf area determined with a planimeter (Model 3100, Li-Cor Inc., Nebraska, USA). After placing the plant in a whole plant pressure chamber, the cut petiole was rinsed three times with distilled water and then blotted with filter paper to remove any contaminating cell debris, then pressure applied (0.05 MPa increments) to the pot until the petiole stub was on the verge of bleeding [22]. Sap was collected for various lengths of time (typically 20 minutes) into pre-weighed eppendorf tubes (allowing calculation of sap flow rate). The whole shoot was then removed, the cut stump was washed with distilled water and then blotted with filter paper to remove any contaminating cell debris, then sap collected with a descending series of overpressures (typically 5) for 1-2 min, to allow analyses of root xylem sap samples that most closely matched whole plant transpiration rate. All samples were collected using a glass capillary tube and immediately transferred to a pre-weighed eppendorf tube sitting in ice. Samples were weighed (to determine sap flow rate), then stored at -80°C prior to measurement of ABA (and metabolite) concentrations either by radio-immunoassay or GC-MS procedures as described below. After sap collection, soil (including roots) was collected and weighed, then oven-dried at 80°C for 5 days and re-weighed, to estimate soil gravimetric water content at harvest. Soil water content generally exceeded 0.3 g g^-1 ^(except in one experiment described above) in all plants harvested, thus the lowest soil matric potential was 100 kPa (calculated from the moisture release curve in [15])

### Separation of ABA precursors, ABA and ABA-GE from xylem sap

Procedures were adapted from [33] to quantify free ABA, the ABA *ex *Adduct II and the ABA *ex *Adduct I, with the development and addition of the capability to also measure ABA *ex *ABA-GE. The ABA released from each of these compounds was quantified as the pentafluoro-benzyl ester (PFBABA).

Side-chain tetradeuteriated ABA (D_4_ABA: 10 ng [2-^2^H, 6-^2^H_3_]ABA from the Plant Biotechnology Institute, National Research Council of Canada, Saskatoon, Saskatchewan S7N 0 W9, Canada) was added to each xylem sap sample followed by sufficient water to make the sample volume up to 0.25 mL. Then an equal volume of 0.02 M cetyltrimethylammonium bromide (CTMABr) was added (0.25 ml, or more if the sap volume was greater than 0.25 mL), followed by 1 ml dichloromethane (DCM): *iso-*propyl alcohol (IPA) = 4: 1 (or 4x the volume of CTMABr added). The phases were mixed gently by tilting the tube back and forth and then the lower organic phase was removed with a disposable glass Pasteur pipette. The addition of DCM: IPA = 4: 1 followed by the mixing and extraction was repeated four times to give a combined organic phase, which contained free ABA and ABA-GE, and which was then evaporated to dryness under nitrogen.

The aqueous phase, which contained Adduct I and Adduct II, then had 10 ng D_4_ABA and one drop of the pH indicator bromophenol blue (BPB: 1% in water) followed by 0.1 M H_2_SO_4 _added dropwise until the solution turned yellow (pH approx. 2). After standing for at least 30 minutes the ABA *ex *Adduct II was extracted three times with at least an equal volume of ether on each occasion, and the combined ether extracts dried over anhydrous Na_2_SO_4_, removed to a clean tube and evaporated under nitrogen. From earlier preliminary investigation, this ABA *ex *Adduct II was calculated to contain a negligible 0.6% of the carried over total ABA-GE, which was not assayed as either ABA *ex *Adduct II or as ABA *ex *ABA-GE.

The aqueous phase remaining after the extraction of the ABA *ex *Adduct II then had 10 ng D_4_ABA added followed by one drop of phenolphthalein (1.0 mM in 40% ethanol) with the subsequent dropwise addition of 0.1 M NaOH to give a final pH of approximately 10. After standing for at least one hour the solution was acidified with 1.0 M H_2_SO_4 _and extracted three times with ether as above. These combined ether extracts were then dried over Na_2_SO_4_, removed and evaporated under nitrogen. This sample contained ABA *ex *Adduct I as well as approximately 0.8% of the carried over total ABA-GE, a small fraction of which may have been assayed as ABA *ex *Adduct I. To correct for quantification, it was estimated that the discarded aqueous phase typically contained approximately 7 to 8% of unrecovered ABA-GE.

The evaporated organic phase from the initial extraction had 0.5 mL H_2_O added prior to mixing, with the solution then having one drop BPB added followed by dropwise acidification with 0.1 M H_2_SO_4_. This was then extracted once with ethyl acetate to give the free ABA, while ABA-GE remained in the aqueous phase. The evaporated ethyl acetate then had 0.5 mL H_2_O added and the solution, which was free of CTMA^+^, was then acidified with 0.1 M H_2_SO_4 _(some BPB was present but a clearer result was obtained if a further drop of BPB was added). The acidified solution was then extracted three times with ether and the combined extracts evaporated. This procedure gave the free ABA, free of ABA-GE and the ion pairing reagent (CTMABr).

The aqueous phase containing ABA-GE had 10 ng D_4_ABA added followed by one tenth the volume of this aqueous phase of 11 M NaOH to give a final concentration of 1.0 M NaOH which, when left for at least one hour, saponified the ABA-GE. When saponification was complete the solution was acidified with 10 M H_2_SO_4 _(sufficient BPB was present from the earlier steps to detect the colour change) and then extracted three times with ether. The combined ether extracts were dried over Na_2_SO_4_, removed to a clean tube and evaporated to dryness.

### Assaying ABA ex Adduct II, ABA ex Adduct I, free ABA and ABA ex ABA-GE

A fresh solution of dimethyl acetamide (DMA): tetramethylammonium hydroxide (TMAOH: 1.1 M in methanol: MeOH) = 5:1 was prepared as was a solution of DMA: pentafluorobenzyl bromide (PFBBr) = 10: 3. Then 10 μL of DMA:TMAOH was added to the sample and this was immediately followed by 10 μl DMA: PFBBr, the solution being mixed immediately. Following this reaction, to confirm that the solution remained alkaline and ensure that esterification of ABA had gone to completion, two further drops of DMA:PFBBr were added. Next, 20 μL H_2_O was added to the alkaline solution followed by 10 μL*n-*butanol and 100 μL hexane. Excess PFBBr and the PFB esters were extracted into the hexane layer which was removed and evaporated. The PFB esters were then purified by solid phase extraction (SPE) on silica columns (AccuBOND 11, Agilent Technologies Ltd, Edinburgh, UK). Columns were attached to a 24-port SPE vacuum manifold (Supelco, Bellefonte, USA) and activated by washing with 2 ml hexane followed by 2 mL dry (molecular sieve) dichloromethane (DCM). Samples were then loaded onto columns in 2 × 100 μL dry DCM and then columns were washed with 1 mL dry DCM to elute any excess PFBBr. The PFB esters were then eluted with 1 ml 15% ether in DCM into clean 1.5 mL GC-MS vials (Crawford Scientific, Lanarkshire, UK) and the eluting solvent evaporated under nitrogen. The PFB esters were then transferred to 100 μL vial inserts (Crawford Scientific, Lanarkshire, UK) with 2 × 50 μL ethyl acetate which was then evaporated prior to the addition of 20 μL ethyl acetate for the quantification of PFBABA by GC-MS.

### Assaying methyl abscisate (MeABA) ex Adduct I and MeABA ex Adduct II

The PFBABA samples from ABA *ex *Adduct I and ABA *ex *Adduct II also contained MeABA as previously observed [6,7]. A MeABA internal standard was therefore prepared from D_4_ABA utilising 1-methyl-3-nitro-1-nitrosoguanidine in an Aldrich MNNG-diazomethane (CH_2_N_2_) generator. Samples of MeD_0_ABA then had 10 ng D_4_ABA added and the PFB esters were synthesised as above so that the treatment of these standards paralleled the treatment of the sap samples analysed in the experiments reported here. This showed that ng PFBD_4_ABA × (ng MeD_0_ABA)^-1 ^= 3.55. This sensitivity ratio was therefore used in quantifying MeABA *ex *Adduct I and MeABA *ex *Adduct II in the experiments reported here.

### Comparison of GC-MS and radioimmunoassay (RIA) determinations

Xylem sap was harvested from the roots and stems of three tomato plants as described above. The total sap volume from each sample was divided in half. Ten nanograms of D_4_ABA was added to one aliquot at the appropriate steps and the four components were then separated and assayed for ABA by the PFB method described above. The second aliquot was used for the determination of ABA by RIA. To minimise the potential risk of interference during ABA:antibody binding, no D_4_ABA or pH indicators were added, but this aliquot was otherwise treated in exactly the same way with the same number of drops of acid or base being added as in the samples for GC-MS analysis. The samples for RIA had any ether remaining from the extraction procedures evaporated and were then made up to 0.5 mL with H_2_O and assayed as described previously [34].

### GC-MS quantification of MeABA and PFBABA

Sample quantification was performed using an Agilent 6890 Network GC connected to a 5973 N Mass Selective Detector (MSD) with electron impact ionisation (70 eV), a 0.25 mm×30 m×0.25 μm HP-5MS column (Agilent, Palo Alto, CA) and a constant helium flow rate of 1.5 mL/min. Samples were loaded onto an Agilent 7683 Series Autosampler and following a pre-injection solvent wash with MeOH to prevent sticking of the syringe plunger and a second wash with ethyl acetate to match the solvent in samples, 5 μL of each sample was injected into an inlet maintained at 250°C and operating in splitless mode. An initial oven temperature of 180°C was maintained for the first 3 minutes then ramped at 10°C min^-1 ^to 280°C, held for 2 minutes and then ramped to 300°C at 20°C min^-1 ^to be held for a final 2 minutes. The MSD transfer line was maintained at 270°C, the MSD quad and source at 150°C, and the ions 141, 278, 281 and 282 (MeABA); and 263, 264, 265, 266 and 267 (PFBABA) were monitored in Selected Ion Monitoring (SIM) mode with a dwell time of 100 milliseconds. The peak area ratio (263:267) was determined and the amount of PFBABA in samples was extrapolated from a previous calibration plot of peak area ratio versus mole ratio (^1^H:^2^H). To determine the amount of MeABA *ex *Adduct I and MeABA *ex *Adduct II, the peak area ratio (278 MeD_0_ABA:267 PFBD_4_ABA) was determined, and corrected using the sensitivity ratio described above.

### Statistics

Genotypic differences in soil water content, leaf water potential and xylem sap constituents were determined by analysis of variance, with Tukey's HSD test (P < 0.05) used to discriminate means.

## Abbreviations

ABA-GE: ABA glucose-ester; AC: (cultivar) Ailsa Craig; BPB: bromophenol blue; *flc*: *flacca*; GC-MS: gas chromatography-mass spectrometry; NCED: 9-*cis*-epoxycarotenoid dioxygenase; *not*: *notabilis*; Ψ_leaf_: leaf water potential; RIA: radioimmunoassay; RR: (cultivar) Rheinlands Ruhm; *sit*: *sitiens*; WT: wild-type

## Competing interests

The authors declare that they have no competing interests.

## Authors' contributions

AGN and ICD conceived the study and drafted the manuscript. ICD grew plants, collected xylem sap samples and conducted radio-immunoassays. AGN and JCT designed and executed the GC-MS procedures to separate forms of ABA. JCT and ICD. All surviving authors read and approved the final manuscript.

## References

[B1] Dodd IC Root-to-shoot signalling: assessing the roles of "up" in the up and down world of long-distance signalling *in planta* Plant Soil 2005 274 251 270 10.1007/s11104-004-0966-0

[B2] Jiang F Hartung W Long-distance signalling of abscisic acid (ABA): the factors regulating the intensity of the ABA signal J Exp Bot 2008 59 37 43 17595196 10.1093/jxb/erm127

[B3] Holbrook NM Shashidhar VR James RA Munns R Stomatal control in tomato with ABA-deficient roots: response of grafted plants to soil drying J Exp Bot 2002 53 1503 1514 10.1093/jexbot/53.373.1503 12021298

[B4] Chen G Lips SH Sagi M Biomass production, transpiration rate and endogenous abscisic acid levels in grafts of *flacca *and wild-type tomato *(Lycopersicon esculentum)* Funct Plant Biol 2002 29 1329 1335 10.1071/PP01263 32688731

[B5] Dodd IC Theobald JC Richer SK Davies WJ Partial phenotypic reversion of ABA-deficient *flacca *tomato (*Solanum lycopersicum*) scions by a wild-type rootstock: normalising shoot ethylene relations promotes leaf area but does not diminish whole plant transpiration rate J Exp Bot 2009 60 4029 4039 10.1093/jxb/erp236 19648172 PMC2755025

[B6] Netting AG Windsor ML Milborrow BV The isolation and identification of the prosthetic group released from a bound form of abscisic acid Plant Growth Regulation 1992 11 327 334 10.1007/BF00024572

[B7] Netting AG Windsor ML Milborrow BV Endogenous biosynthetic precursors of (+)-abscisic acid. 3. Incorporation of H_2 _from (H_2_O)-H-2 and O-18 from O-18(2) into precursors Aust J Plant Physiol 1997 24 175 184 10.1071/PP96057

[B8] Taylor IB Sonneveld T Bugg TDH Thompson AJ Regulation and manipulation of the biosynthesis of abscisic acid, including the supply of xanthophyll precursors J Plant Growth Regul 2005 24 253 273

[B9] Burbidge A Grieve TM Jackson A Thompson A McCarty DR Taylor IB Characterization of the ABA-deficient tomato mutant *notabilis *and its relationship with maize Vp14 Plant J 1999 17 427 431 10.1046/j.1365-313X.1999.00386.x 10205899

[B10] Harrison E Burbidge A Okyere JP Thompson AJ Taylor IB Identification of the tomato ABA-deficient mutant sitiens as a member of the ABA-aldehyde oxidase gene family using genetic and genomic analysis Plant Growth Regul 2011 64 301 309 10.1007/s10725-010-9550-1

[B11] Sauter A Dietz K-J Hartung W A possible stress physiological role of abscisic acid conjugates in root-to-shoot signalling Plant Cell Environ 2002 25 223 228 10.1046/j.1365-3040.2002.00747.x 11841665

[B12] Dietz KJ Wichert K Sauter A Messdaghi D Hartung W Characterisation of an extracellular B-glucosidase in barley involved in the hydrolysis of ABA glucose conjugate in leaves J Exp Bot 2000 51 937 944 10.1093/jexbot/51.346.937 10948220

[B13] Else MA Taylor JM Atkinson CJ Anti-transpirant activity in xylem sap from flooded tomato (*Lycopersicon esculentum *Mill.) plants is not due to pH-mediated redistributions of root- or shoot-sourced ABA J Exp Bot 2006 57 3349 3357 10.1093/jxb/erl099 16940038

[B14] Dodd IC Measuring and modeling xylem ABA concentration ([X-ABA]) in tomato plants exposed to deficit irrigation (DI) and partial rootzone drying (PRD) Acta Horticulturae 2008 792 225 231

[B15] Dodd IC Egea G Watts C Whalley WR Root water potential integrates discrete soil physical properties to influence ABA signalling during partial rootzone drying J Exp Bot 2010 61 3543 3551 10.1093/jxb/erq195 20591896

[B16] Plauborg F Abrahamsen P Gjettermann B Mollerup M Iversen BV Liu F Andersen MN Hansen S Modelling of root ABA synthesis, stomatal conductance, transpiration and potato production under water saving irrigation regimes Agr Water Manag 2010 98 425 439 10.1016/j.agwat.2010.10.006

[B17] Taylor IB Tarr AR Phenotypic interactions between abscisic acid deficient tomato mutants Theor Appl Genet 1984 68 115 119 10.1007/BF00252325 24258952

[B18] Dodd IC Soil moisture heterogeneity during deficit irrigation alters root-to-shoot signalling of abscisic acid Funct Plant Biol 2007 34 439 448 10.1071/FP07009 32689371

[B19] Else MA Davies WJ Whitford PN Hall KC Jackson MB Concentrations of abscisic acid and other solutes in xylem sap from root systems of tomato and castor-oil plants are distorted by wounding and variable sap flow rates J Exp Bot 1994 45 313 323

[B20] Jachetta JJ Appleby AP Boersma L Use of the pressure-vessel to measure concentrations of solutes in apoplastic and membrane-filtered symplastic sap in sunflower leaves Plant Physiol 1986 82 995 999 10.1104/pp.82.4.995 16665180 PMC1056247

[B21] Jokhan AD Harink RJ Jackson MB Concentration and delivery of abscisic acid in xylem sap are greater at the shoot base than at a target leaf nearer to the shoot apex Plant Biology 1999 1 253 260 10.1111/j.1438-8677.1999.tb00251.x

[B22] Li B Feng Z Xie M Sun M Zhao Y Liang L Liu G Zhang J Jia W Modulation of the root-sourced ABA signal along its way to the shoot in *Vitis riparia × Vitis labrusca *under water deficit J Exp Bot 2011 62 1731 1741 10.1093/jxb/erq390 21131549

[B23] Schurr U Xylem sap sampling - new approaches to an old topic Trends Plant Sci 1997 3 293 298

[B24] Tiekstra AE Else MA Jackson MB External pressures based on leaf water potentials do not induce xylem sap to flow at rates of whole plant transpiration from roots of flooded or well-drained tomato and maize plants. Impact of shoot hydraulic resistances Ann Bot 2000 86 665 674 10.1006/anbo.2000.1193

[B25] Chen G Fu XP Lips SH Sagi M Control of plant growth resides in the shoot, and not in the root, in reciprocal grafts of *flacca *and wild-type tomato (*Lycopersicon esculentum*) in the presence and absence of salinity stress Plant Soil 2003 256 205 215

[B26] Neill SJ Horgan R Abscisic acid production and water relations in wilty tomato mutants subjected to water deficiency J Exp Bot 1985 36 1222 1231 10.1093/jxb/36.8.1222

[B27] Jones HG Sharp CS Higgs KH Growth and water relations of wilty mutants of tomato (*Lycopersicon esculentum *Mill.) J Exp Bot 1987 38 1848 1856 10.1093/jxb/38.11.1848

[B28] Cornish K Zeevaart JAD Phenotypic expression of wild-type tomato and three wilty mutants in relation to abscisic acid accumulation in roots and leaflets of reciprocal grafts Plant Physiol 1988 87 190 194 10.1104/pp.87.1.190 16666101 PMC1054723

[B29] Mulholland BJ Hussain A Black CR Taylor IB Roberts JA Does root-sourced ABA have a role in mediating growth and stomatal responses to soil compaction in tomato (*Lycopersicon esculentum*)? Physiol Plant 1999 107 267 276 10.1034/j.1399-3054.1999.100303.x

[B30] Sagi M Fluhr R Lips SH Aldehyde oxidase and xanthine dehydrogenase in a *flacca *tomato mutant with deficient abscisic acid and wilty phenotype Plant Physiol 1999 120 571 577 10.1104/pp.120.2.571 10364409 PMC59296

[B31] Mulholland BJ Thompson AJ Jackson AC Taylor IB McKee JMT Can ABA mediate salinity root stress in tomato? Environ Exp Bot 2003 50 17 28 10.1016/S0098-8472(02)00110-7

[B32] Linforth RST Bowman WR Griffin DA Marples BA Taylor IB 2-trans-ABA alcohol accumulation in the wilty tomato mutants *flacca *and *sitiens* Plant Cell Environ 1987 10 599 606

[B33] Duffield PH Netting AG Methods for the quantitation of abscisic acid and its precursors from plant tissues Anal Biochem 2001 289 251 259 10.1006/abio.2000.4943 11161319

[B34] Quarrie SA Whitford PN Appleford NEJ Wang TL Cook SK Henson IE Loveys BR A monoclonal antibody to (S)-abscisic acid: its characterisation and use in a radioimmunoassay for measuring abscisic acid in crude extracts of cereal and lupin leaves Planta 1988 173 330 339 10.1007/BF00401020 24226540

